# Maternal Deprivation Influences Pup Ultrasonic Vocalizations of C57BL/6J Mice

**DOI:** 10.1371/journal.pone.0160409

**Published:** 2016-08-23

**Authors:** Xiaowen Yin, Ling Chen, Yong Xia, Qunkang Cheng, Jiabei Yuan, Yan Yang, Zhaoxin Wang, Haojie Wang, Jianshu Dong, Yuqiang Ding, Xudong Zhao

**Affiliations:** 1 Department of Psychosomatic Medicine, East Hospital, Tongji University School of Medicine, Shanghai, China; 2 Key Laboratory of Arrhythmias, Ministry of Education, East Hospital, Tongji University School of Medicine, Shanghai, China; 3 Department of Psychiatry, The Seventh Hospital of HangZhou, Zhejiang, China; 4 Department of Anatomy and Neurobiology, Tongji University School of Medicine, Shanghai, China; 5 Department of Entomology and Plant Pathology, University of Tennessee, Knoxville, Tennessee, United States of America; 6 Shanghai Health Education Institute, Shanghai, China; Universidade do Estado do Rio de Janeiro, BRAZIL

## Abstract

Maternal deprivation (MD) is frequently used as an early life stress model in rodents to investigate behavioral and neurological responses under stressful conditions. However, the effect of MD on the early postnatal development of rodents, which is when multiple neural systems become established, is rarely investigated due to methodological limitations. Ultrasonic vocalizations (USVs) are one of the few responses produced by neonatal rodents that can be quantitatively analyzed, and the quantification of USVs is regarded as a novel approach to investigate possible alterations in the neurobehavioral and emotional development of infant rodents under stress. To investigate the effect of MD on pup mice, we subjected C57BL/6J mice to MD and recorded the USVs of pups on postnatal days 1, 3, 7, 8, and 14. To determine whether the effect of MD on USVs was acute or cumulative, pre- and post-separation USV groups were included; sex differences in pup USV emission were also investigated. Our results suggest that (i) USV activity was high on postnatal days 3–8; (ii) the MD effect on USVs was acute, and a cumulative effect was not found; (iii) the MD mice vocalized more and longer than the controls at a lower frequency, and the effect was closely related to age; and (iv) female pups were more susceptible than males to the effect of MD on USV number and duration between postnatal days 3–8.

## Introduction

Early adversity, such as parental loss, neglect or abuse, can increase the risk of mental disorders later in life [1,2], as demonstrated by previous animal studies of non-human primates and rodents. For example, exposure to a stressful environment during the neonatal period has been shown to induce persistent changes in emotional behaviors in adulthood [3–5] and to influence the brain systems that are involved in regulating stress responsiveness [6–9]. Maternal deprivation (MD), in which pups are separated from the dam and littermates for a given period of time (e.g.,1-24h) over a number of postnatal days (e.g., P1-14) [10,11], is a stressful procedure for rodents that can be used to investigate behavioral and neurobiological responses to early adversity [4,12]. To the best of our knowledge, prior studies that utilized MD (acute or repeated MD) have mainly focused on the long-term effects of MD on adolescent or adult animals, and ample evidence regarding the influence has been obtained. For instance, adult rats that experienced repeated MD in early life showed enhanced vulnerability to stress, as demonstrated by an increase in depressive behaviors, such as a lower sucrose preference in a sucrose preference test and a longer immobility time in a forced swimming test [13]. Benner suggested that early deprivation can induce competitive subordinance and impaired reversal learning with altered functions of the limbic and frontal cortices in adult C57BL/6 mice [14]. However, the effect of MD in early life, particularly the neonatal days during which multiple neural systems are established [15,16], has rarely been investigated, which may be partially due to the lack of parameters that can be quantitatively analyzed in very young rodents.

Recently, ultrasonic vocalizations (USVs) have received increasing attention as a potential method for investigating the development of communication and emotion in rodents, even when they are very young [17,18]. Rodents can emit and perceive USVs at frequencies above the human hearing threshold of 20 kHz throughout their lifespan [19]. Isolation-induced USVs are whistle-like sounds that are emitted by pups when they are separated from their mother and littermates; these USVs are typically characterized by a frequency of 30–90 kHz and a duration of 10–200 ms [20]. Evidence has shown that pup USVs serve an important communicative function in infant-mother interactions; they can induce maternal behaviors such as nest building, pup retrieval and nursing [19,21,22]. Past studies aimed at characterizing USV emissions under stress revealed that the rate of USVs (USV number) can be influenced by environmental stimuli; this measure has thus been thought to reflect the affective state of rodent pups [23]. For example, Zimmerberg et al. reported that the USV rate of isolated rat pups was significantly lower than that of controls [24], and it was shown that USV rate could be reduced by the administration of anti-anxiety agents such as allopregnanolone [25]. In mandarin vole pups, repeated early deprivation has been reported to decrease the number of calls [26]. However, the USV responses of mice to MD in early life are not yet well understood.

As mentioned above, many questions about the effect of MD on mice remain to be investigated. For example, does the MD paradigm affect very young mice? How effective is it in eliciting mouse USVs? Moreover, due to differences in the adult behaviors of male and female mice, we also wanted to determine whether there are sex differences in pup USV emissions when they are exposed to isolation stress. The C57BL/6 mouse, which is commonly used to generate transgenic or knockout models, is the best choice for neurobiological and behavioral studies [27,28]. To begin to answer some of the questions posed above, we subjected C57BL/6J pups to MD in order to explore its effects on the isolation-induced USVs, including the type of MD effect (acute or cumulative) and sex differences in USV emission.

## Materials and Methods

### Animals

A total of 20 female and 10 male C57BL/6J mice (6 weeks of age) were obtained from the Laboratory Animal Services Center of Tongji University. The mice were habituated to the breeding facility for two weeks prior to mating. Each male was housed with two females in a Plexiglas cage (17.5 cm X 24.5 cm X 12.5 cm) under standard conditions (room temperature 24±1°C, humidity 50±5%), with food and water provided ad libitum and a 12-h light/dark cycle (lights off at 19:00). Visibly pregnant dams were moved to individual cages for parturition and breeding; all the dam were pluriparous. The 20 female mice were not bred at the same time. Because litter size can be a confounding factor for USV developmental variables [29], litters with fewer than six offspring were excluded in our study, and all litters used were demonstrated to not be significantly different in terms of litter size (P = 0.642). In total, 179 pups from 26 litters were equally and randomly allocated to the different rearing conditions described below. Procedures involving animals were performed in strict accordance with the National Institutes of Health Guide for the Care and Use of Laboratory Animals and Chinese legislation on the use and care of laboratory animals. Our study was approved by the Animal Care and Use Committee of Tongji University, where the studies were conducted (Permit Number: TJmed-013-060).

### Maternal deprivation

The morning that pups were found in the nest was designated P0 [30]. On P0, the pups were sexed by anogenital distance [31] and standardized to foster litters composed of 6–10 pups, at a 1:1 male/female ratio if possible [26,32]. Each litter was randomly assigned to one of three rearing conditions. (1) Animal facility-reared (AFR) litters served as a control (n = 35); AFR litters were left in the nest with dams except for the 5 min of USV detection on USV test days. (2) Pups in the MD180 group (n = 70) were separated for 180 min per day from 12:00–15:00. (3) Pups in the MD360 group (n = 74) received 360 min of separation daily from 9:00–15:00. The MD paradigm was conducted from P1-14, during which the pups were removed from their dam and littermates and individually isolated in plastic cups with cotton at the bottom and a heating blanket maintained at 34°C [33,34]. On P2 and P8, the bedding in the home cages of the three groups was replaced with fresh bedding.

### USV detection

Pup USVs were recorded with a Med Associates Ultrasonic Vocalization Detector (ANL-937-1, Med Associates Inc., Florida, USA) that was attached to a unidirectional microphone in the top of the test chamber, which was placed in a sound-attenuated box (Med Associates Inc., Florida, USA). Each USV recording session lasted for 5 min. Before the start of the recording session, the pup was first placed in the center of the chamber floor for 20 s of habituation. A towel was placed at the bottom of the chamber to reduce the amplitude of noises produced by pup movement. The towel was changed between recordings of different litters to prevent odor transfer. The USV detector scanned the ultrasonic frequencies every 30 ms and recorded the amplitude of sound at each frequency between 20 and 100 kHz. The detector was connected to a computer, and the data were acquired with MED USV Application Software SOF-937-1 [35].

### Pre- and post-separation USV groups

Each pup in the AFR group was individually removed from its home cage and placed in the chamber. The pup’s USV production was measured for 5 min, and the pup was then reintroduced to the nest. The pups’ tails were colored with permanent marker pens to distinguish AFR pups.

**Fig 1:**
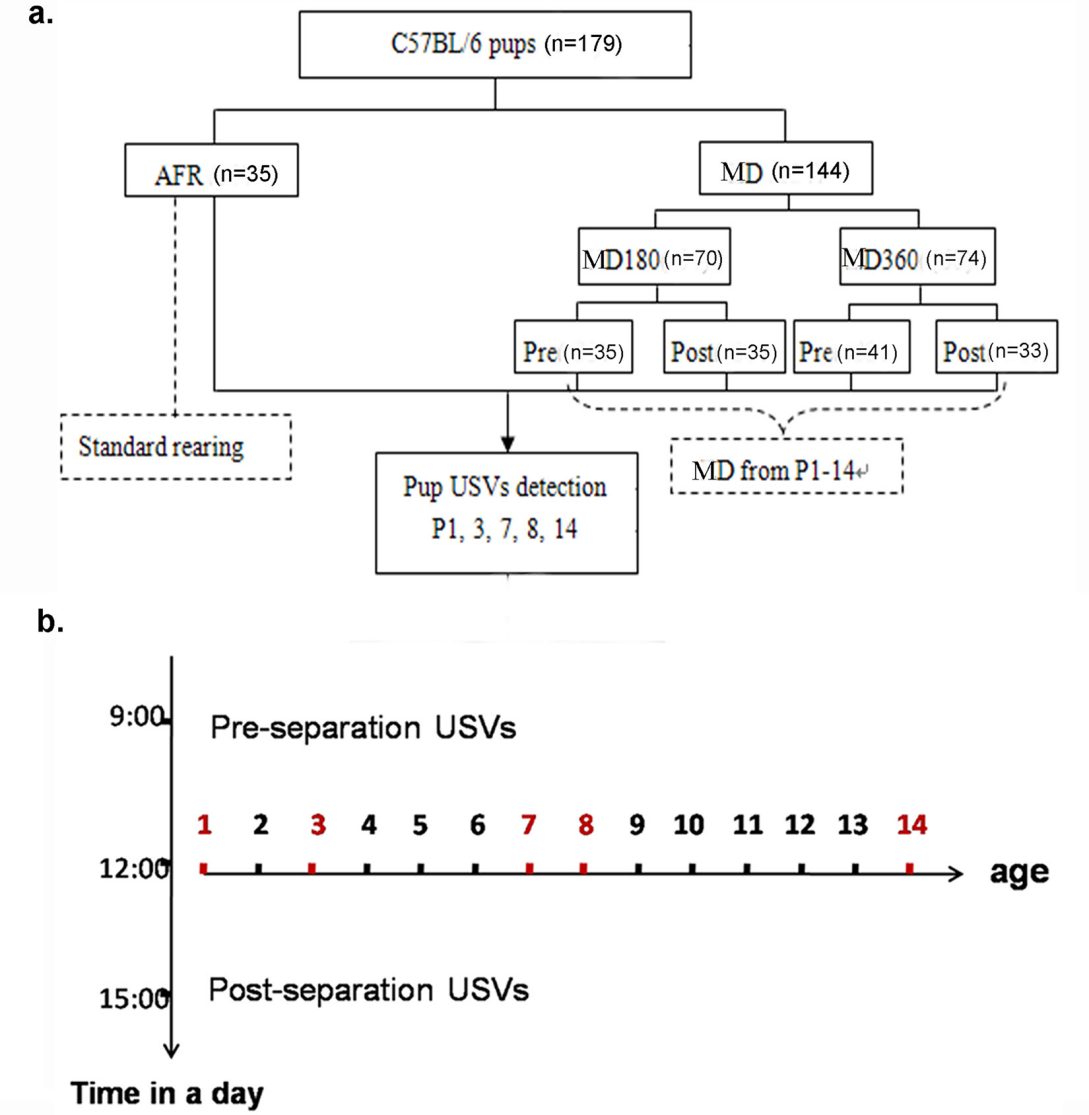
Flow chart of the experimental design. a. The AFR group served as a control with standard rearing; MD refers to the experimental groups, including MD180 and MD360. The pups in the MD180 and MD360 groups were further subdivided into Pre (pre-separation USV) and Post (post-separation USV) groups; five groups (AFR, MD180 Pre, MD180 Post, MD360 Pre, and MD360 Post)were included in the study; b. Mice in the MD groups were separated from their dam and littermates from P1-P14;on P1, P3, P7, P8 and P14, all pups were subjected to USV recording for 5 min. On the testing days, MD180 Pre and MD360 Pre mice were first detected for USVs and then subjected to MD, while MD180 Post and MD360 Post mice were isolated before USV detection.

To investigate whether the effect of MD was acute or cumulative, the pups in the MD180 and MD360 groups were further divided into two subgroups (MD180 Pre and MD180 Post; MD360 Pre and MD360 Post). The pups in the post-separation USV groups (MD180 Post, MD360 Post)were first subjected to MD and then subjected to 5 min of USV detection before they were reintroduced to the home cage. To investigate the possibility of a cumulative effect of MD on USVs, i.e., whether previous MD experience could affect USV emissions during a later evaluation period, pre-separation USV groups (MD180 Pre, MD360 Pre) were also included. For the pups in these groups, USVs were recorded first, and then, the pups were subjected to MD. In this way, five groups were included in our study: AFR (n = 35; five litters: mean number of pups = 7.8; maximum = 8), MD180 Pre (n = 35; five litters: mean number of pups = 7.4; maximum = 9), MD180 Post (n = 35; five litters: mean number of pups = 7; maximum = 8), MD360 Pre (n = 41; six litters: mean number of pups = 7.17; maximum = 9) and MD360 Post (n = 33; five litters: mean number of pups = 7; maximum = 8). All pups were subjected to USV recording on P1, P3, P7, P8 and P14 (Fig 1).

### USV analysis

USV analysis was performed according to previously described methods [35]. The data were analyzed with the “MED-USV.xls” macro for Microsoft Excel. The data output was examined in the form of 3D graphs, where the x-axis was frequency (kHz), the y-axis was amplitude (dB), and the z-axis was time (s). According to the 3D graph, the data were categorized into USVs and other types of sounds (i.e., sounds due to pup movements in the chamber). Only USVs were selected for data analysis. Individual USVs that occurred within 30 ms of one another were considered part of the same call. The USV acoustic features assessed included the USV number, the total duration in 5 min and the average peak frequency. The average peak frequency was expressed as the average of the frequency that corresponded to the maximal amplitude within a call [33]. If an animal did not emit USVs, its data were used only in the USV number and total duration analysis.

### Statistical analysis

All USV data should be transformed to a standardized normal distribution before statistical analysis. We adopted the mixed effect model for the repeated measured data. The PROC MIXED procedure in SAS 9.2 was used for a global assessment of the effect of MD, age and the interaction between MD and age. The CONTRAST statement was used to compare the average level of each variable between MD and age after controlling for the influence of age and MD. The CONTRAST statement provides a mechanism for obtaining custom hypothesis tests (http://www.sas.com/zh_cn/home.html), so it enables us to finish all the pair wise comparisons using the random error and type I error is not necessary corrected in the model.

A P-value of <0.05 was considered significant. The results are presented as the mean±standard error of the mean (SEM). Graphs were generated using GraphPad Prism 6.0.

## Results

### Developmental trajectory of USVs in AFR pups

**Fig 2:**
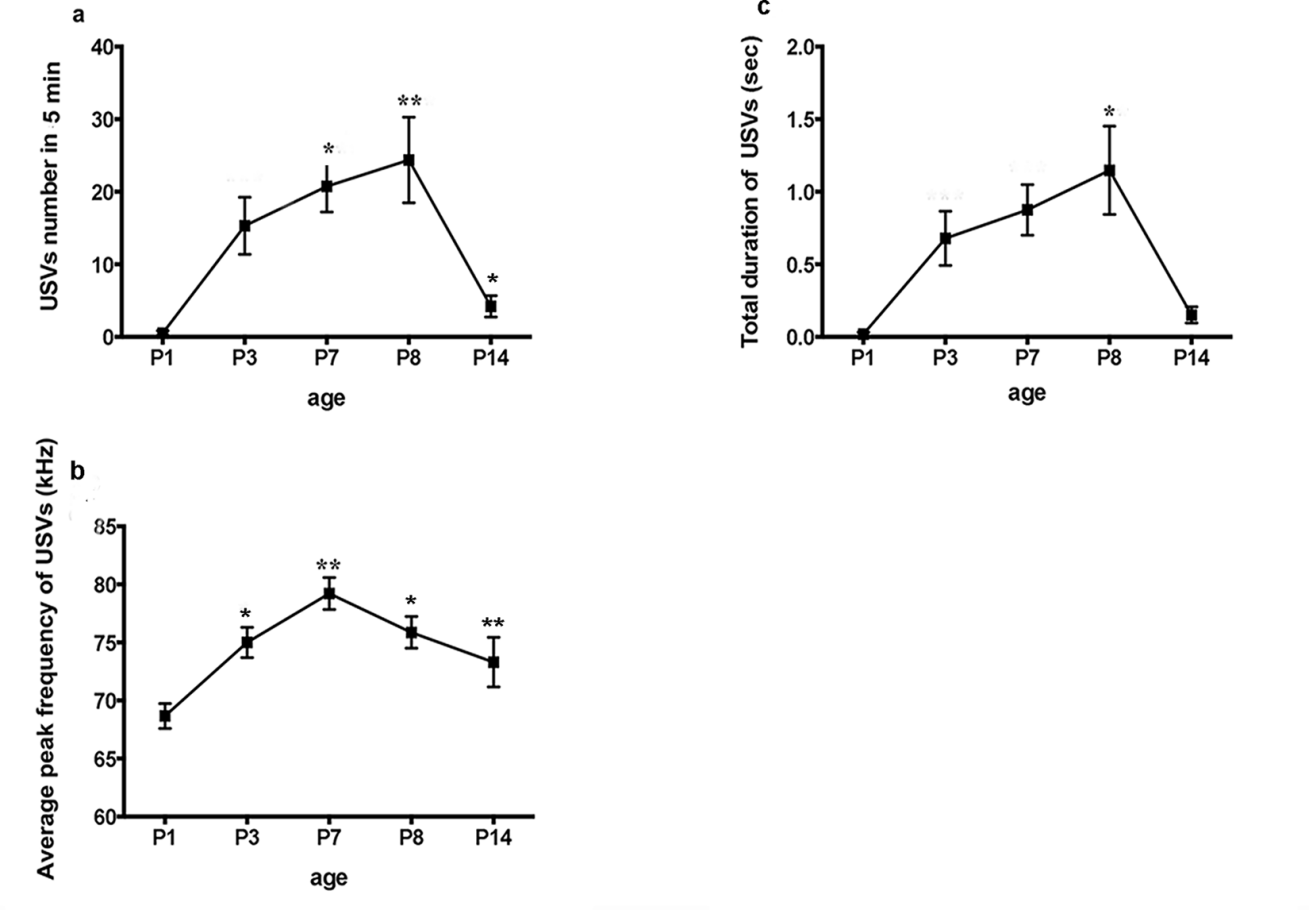
Developmental trajectory of USVs in AFR mice. Age significantly affected (a) USV number and (c) the average peak frequency of USVs. A significant increase in USV number was found on P7 and P8 compared to the corresponding values on P1; the USV number on P8 was more significant than that on P14. The average peak frequency was significantly higher on P7 and P8 than on P1and P3, and USV frequency on P7 was higher than P14. An effect of age was not found for USV duration (b), but the duration of calls on P8 was significantly longer than that on P1. *P<0.05, **P<0.01.

USVs of AFR mice were used to investigate the developmental profile of USVs in C57BL/6J pups without maternal deprivation. We observed a very low number of USVs on P1. The number began to increase on P3, peaked on P8, and then dramatically decreased, nearing 0 on P14. Statistical analysis showed a significant effect of age on USV number (F = 3.02, P = 0.0173), with post hoc tests revealing an increase at P7 and P8 compared to the corresponding values at P1 and P14; in contrast, no difference was found between P3, P7 and P8 (Table 1A) (Fig 2A). Age also affected the average peak frequency (F = 3.53, P = 0.0076), which was significantly higher on P7 than on P1, P3, and P14, and no difference was found between P7 and P8 (Table 1B) (Fig 2B). However, an effect of age was not found in terms of USV duration (F = 1.60, P = 0.1738), although the duration on P8 was longer than that on P1 (Table 1C) (Fig 2C). Our data suggest that the age effect was significant for USV number and average peak frequency; these parameters all peaked between P3 and P8.

**Table 1:** Effect of age on USV in AFR pups.

**a. USV number**				
Group Age	Results of time comparison		Results of overall time comparison	
	*F*_(1,690)_	*P*	*F*_(4,690)_	*P*
AFR P1 vs.P3	2.43	0.1198	3.02	0.0173*
P1 vs.P7	5.02	0.0254 *		
P1 vs.P8	9.45	0.0022 **		
P1 vs.P14	0.38	0.5352		
P3 vs.P7	0.47	0.4952		
P3 vs.P8	2.33	0.1270		
P3 vs.P14	0.88	0.3490		
P7 vs.P8	0.72	0.3951		
P7 vs.P14	2.62	0.1058		
P8 vs.P14	6.04	0.0142 *		
**b. USV frequency**				
Group Age	Results of time comparison		Results of overall time comparison	
	*F*_(1,690)_	*P*	*F*_(4,690)_	*P*
AFR P1 vs.P3	3.04	0.0821	3.53	0.0076**
P1 vs.P7	9.74	0.0019**		
P1 vs.P8	5.49	0.0196*		
P1 vs.P14	1.87	0.1724		
P3 vs.P7	4.29	0.0388 *		
P3 vs.P8	0.75	0.3878		
P3 vs.P14	0.29	0.5900		
P7 vs.P8	1.67	0.1968		
P7 vs.P14	7.06	0.0082 **		
P8 vs.P14	2.07	0.1507		
**c. USV duration**				
Group Age	Results of time comparison		Results of overall time comparison	
	*F*_(1,690)_	*P*		*F*_(1,690)_
AFR P1 vs.P3	1.39	0.2394	1.60	0.1738
P1 vs.P7	2.33	0.1277		
P1 vs.P8	4.94	0.0266*		
P1 vs.P14	0.13	0.7175		
P3 vs.P7	0.12	0.7283		
P3 vs.P8	1.11	0.2921		
P3 vs.P14	0.66	0.4151		
P7 vs.P8	0.50	0.4783		
P7 vs.P14	1.35	0.2452		
P8 vs.P14	3.47	0.628		

*P<0.05

** P<0.01

### Effect of MD on post-separation USVs

**Fig 3:**
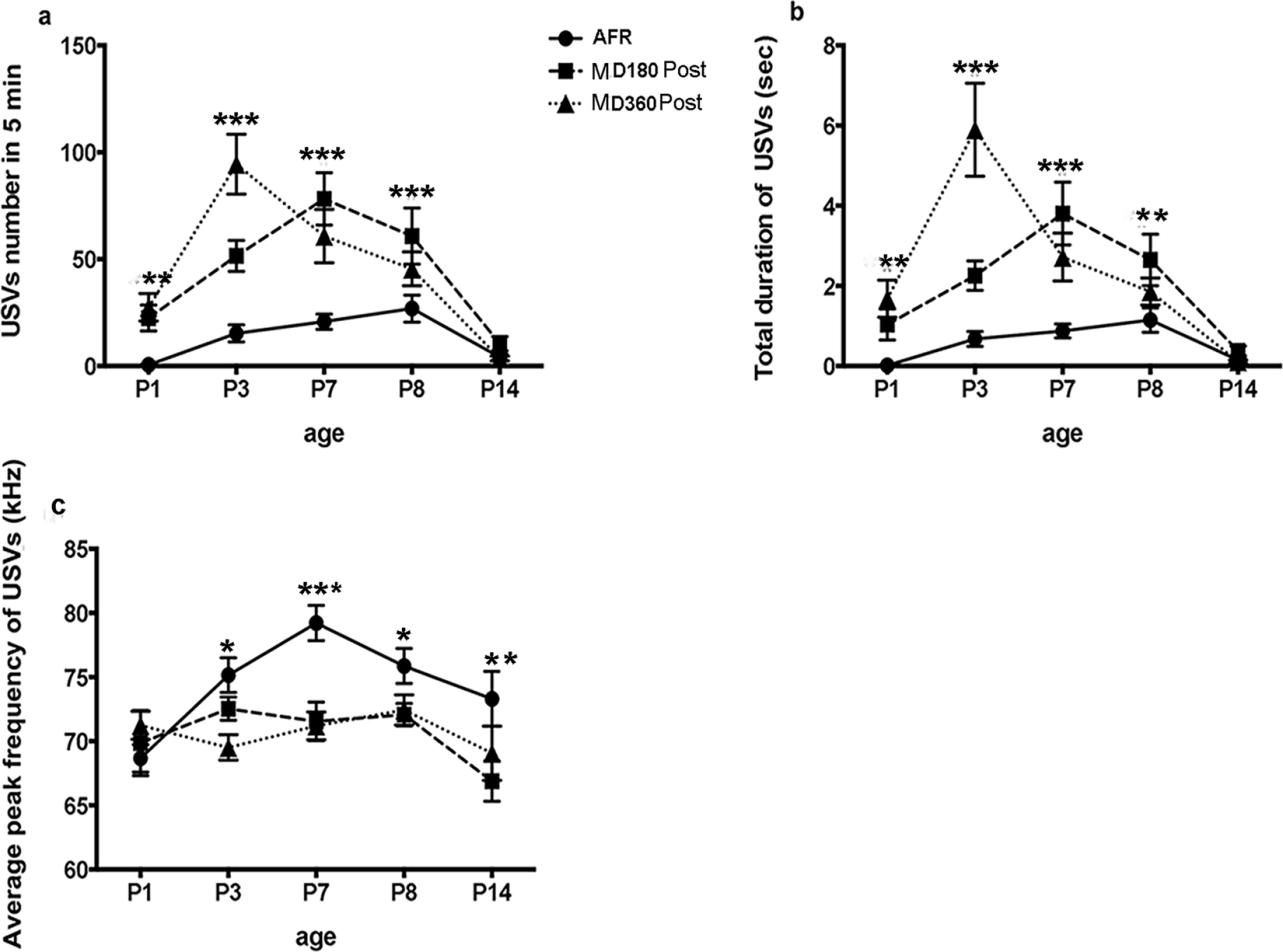
Age XMD effect on post-separation USVs. The interaction effect between age and MD was significant for (a) USV number, (b) the total duration of USVs over a 5-min period and (c) the average frequency of USVs. The MD (MD180 and MD360) mice called more frequently than the AFR mice on P1, P3, P7 and P8,whereas no difference was found on P14 (a); MD180 Post pups called for longer duration than AFR mice on P3, P7and P8, while MD360 Post pups vocalized longer than AFR pups on P1, P3 and P7 (b). For USV frequency, MD180 Post pups called at a lower frequency than AFR on P7, P8 and P14, whereas the MD360 Post mice called with a lower frequency than AFR pups on P3 and P7. *P<0.05, **P<0.01.

The results showed significant differences in USV number between AFR, MD180 Post and MD360 Post pups on P1, P3, P7 and P8, while no difference was detected on P14 (Table 2A). USV duration exhibited a similar trend in conjunction with USV number, with significant differences detected on P1, P3, P7 and P8 and no significant difference detected on P14 (Table 2B). For USV frequency, differences between three groups were found on P3, P7, P8 and P14, while no difference was found on P1 (Table 2C). These results indicate that the MD mice called more frequently and for longer durations than the AFR mice on P1, P3, P7 and P8; MD180 Post and MD360 Post mice called at a lower frequency than AFR mice on P3, P7, P8 and P14. Our data suggest significant changes in USV emission after MD, and the effect of MD was closely related to age (Fig 3).

**Table 2:** Effect of MD on USV of AFR, MD180 Post and MD360 Post pups.

**a. USV number**		
Age Group	Results of group comparison	
	*F*_(1,690)_	*P*
P1 AFR vs. MD180 Post	5.63	0.0179*
AFR vs. MD360 Post	8.82	0.0031**
MD180 Post vs. MD360 Post	0.40	0.5281
P3 AFR vs. MD180 Post	16.96	<0.0001***
AFR vs. MD360 Post	68.32	<0.0001***
MD180 Post vs. MD360 Post	17.71	<0.0001***
P7 AFR vs. MD180 Post	39.31	<0.0001***
AFR vs. MD360 Post	19.48	<0.0001***
MD180 Post vs. MD360 Post	3.11	0.0782
P8 AFR vs. MD180 Post	12.66	0.0004***
AFR vs. MD360 Post	4.51	0.0341*
MD180 Post vs. MD360 Post	1.94	0.1642
P14 AFR vs. MD180 Post	0.31	0.5792
AFR vs. MD360 Post	0.13	0.7148
MD180 Post vs. MD360 Post	0.82	0.3665
**b. USV duration**		
Age Group	Results of group comparison	
	*F*_(1,690)_	*P*
P1 AFR vs. MD180 Post	2.96	0.0856
AFR vs. MD360 Post	9.87	0.0018 **
MD180 Post vs. MD360 Post	2.09	0.1488
P3 AFR vs. MD180 Post	9.88	0.0017 **
AFR vs. MD360 Post	85.73	<0.0001 ***
MD180 Post vs. MD360 Post	37.96	<0.0001 ***
P7 AFR vs. MD180 Post	31.17	<0.0001 ***
AFR vs. MD360 Post	12.58	0.0004 **
MD180 Post vs. MD360 Post	3.82	0.0511
P8 AFR vs. MD180 Post	7.32	0.0070 **
AFR vs. MD360 Post	2.00	0.1578
MD180 Post vs. MD360 Post	1.59	0.2080
P14 AFR vs. MD180 Post	0.11	0.7427
AFR vs. MD360 Post	0.06	0.8138
MD180 Post vs. MD360 Post	0.31	0.5797
**c. USV frequency**		
Age Group	Results of group comparison	
	*F*_(1,690)_	*P*
P1 AFR vs. MD180 Post	0.06	0.8121
AFR vs. MD360 Post	0.41	0.5231
MD180 Post vs. MD360 Post	0.40	0.5258
P3 AFR vs. MD180 Post	0.74	0.3904
AFR vs. MD360 Post	4.76	0.0296 *
MD180 Post vs. MD360 Post	2.11	0.1470
P7 AFR vs. MD180 Post	14.43	0.0002 ***
AFR vs. MD360 Post	13.00	0.0003 ***
MD180 Post vs. MD360 Post	0.01	0.9086
P8 AFR vs. MD180 Post	4.97	0.0262 *
AFR vs. MD360 Post	3.28	0.0710
MD180 Post vs. MD360 Post	0.17	0.6796
P14 AFR vs. MD180 Post AFR vs. MD360 Post	7.532.94	0.0063 **0.0874
MD180 Post vs. MD360 Post	0.84	0.3592

*P<0.05

** P<0.01

*** P<0.001

### Effect of MD on pre-separation USVs

**Fig 4:**
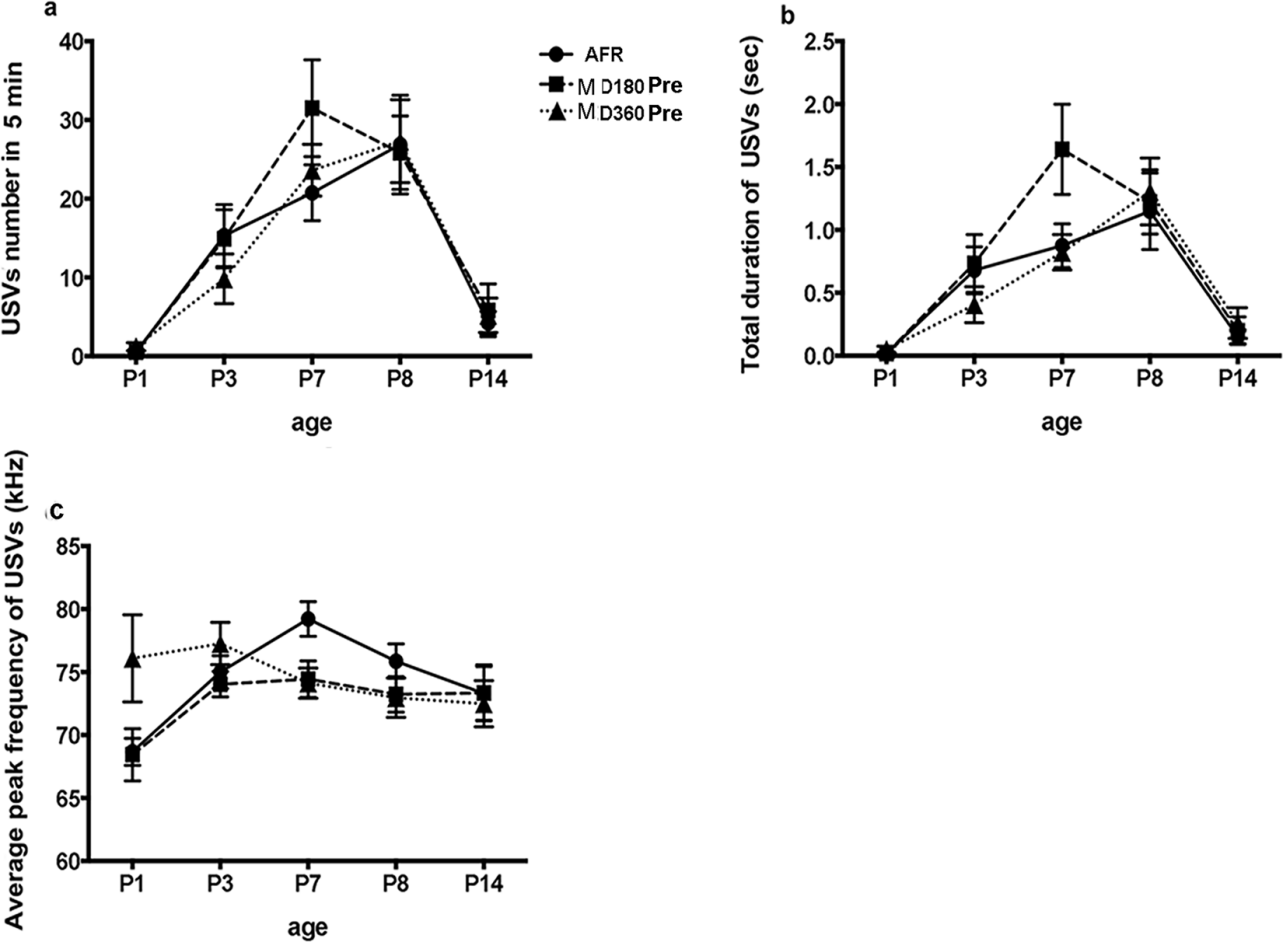
Age X MD effect on pre-separation USVs. The interaction effect between age and MD on (a) USV number, (b) total duration over a 5-min period and (c) the average peak frequency of USVs was not significant.

No significant differences were found between the USVs of AFR, MD180 Pre and MD360 Pre mice (S1 Table). The trajectory of USV development (interaction of age X MD) in the AFR, MD180 Pre and MD360 Pre pups was similar from P1-P14 (Fig 4). Our data indicate that MD did not influence pre-separation USVs.

### Comparison between pre- and post-separation USVs

**Fig 5:**
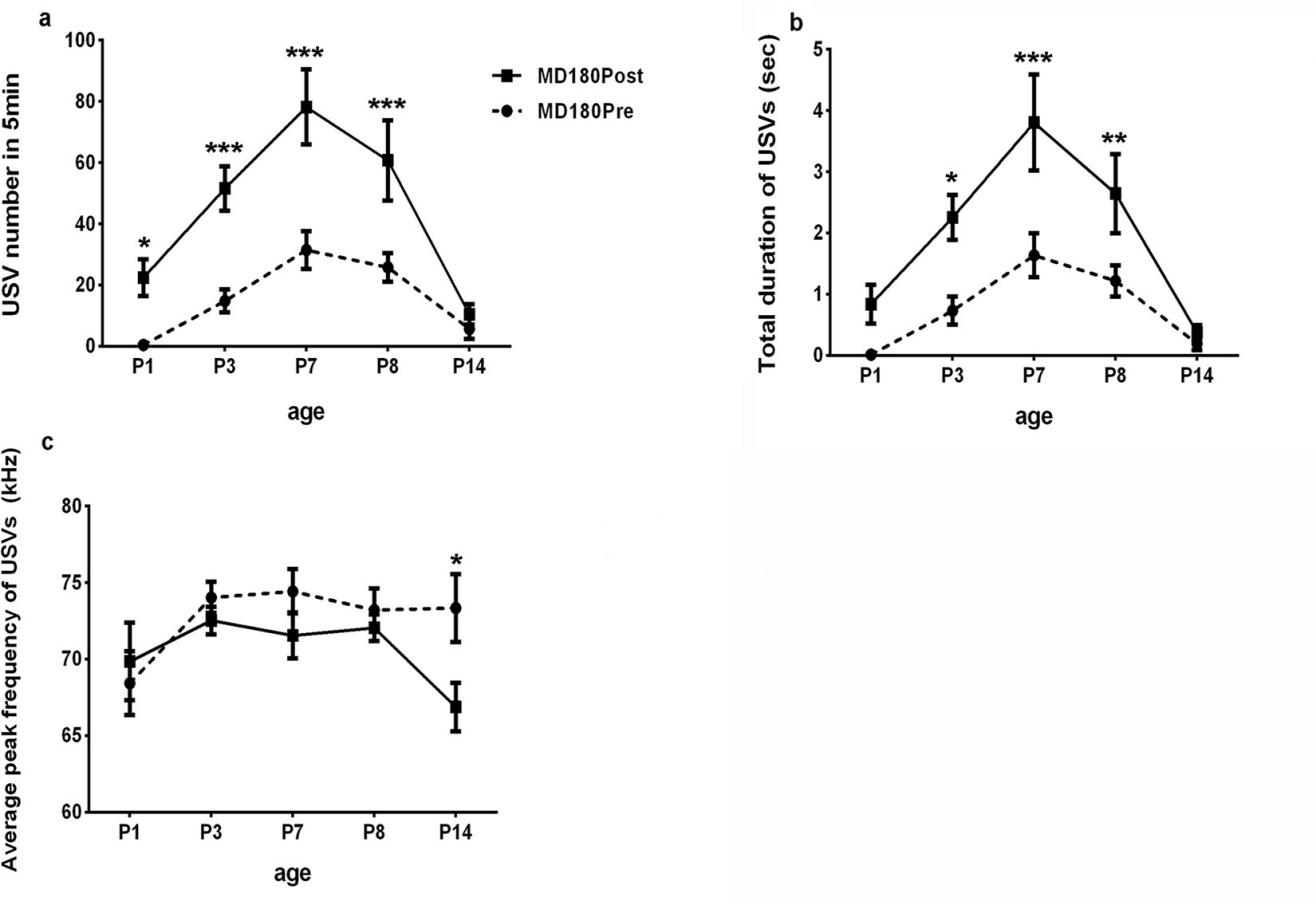
Comparison between MD180 Pre and MD180 Post. a. USV number in 5 min. A significant increase was found in post-separation USVs on P1, P3, P7 and P8. b. Total duration of USVs. The mice in the MD180 Post group vocalized longer than the mice in the MD180 Pre group on P3, P7 and P8. c. Average USV frequency. A difference in USV frequency was found between the MD180 Pre and MD180 Post groups only on P14. *P<0.05, **P<0.01, *** P<0.0001.

**Fig 6:**
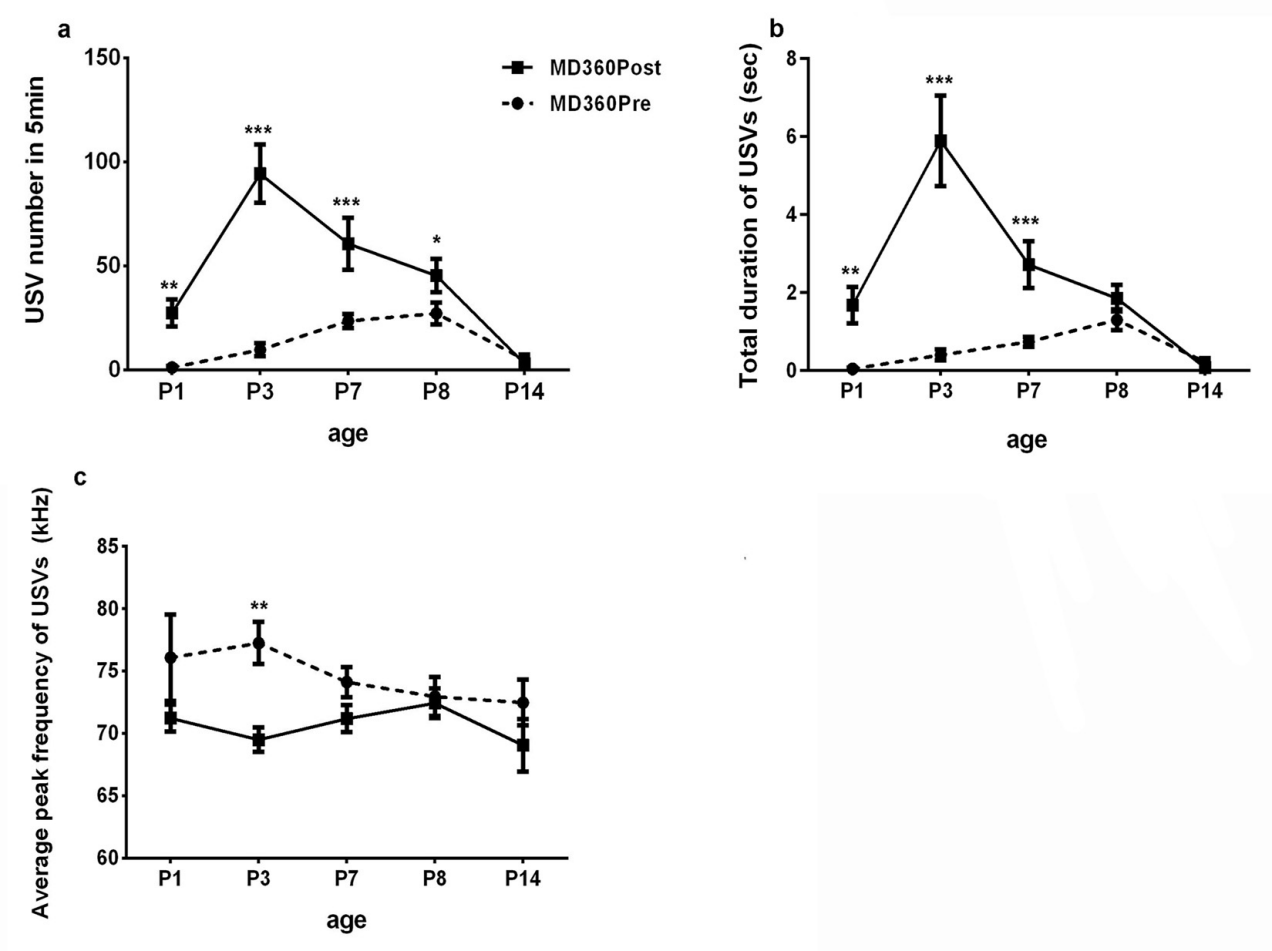
Comparison between MD360 Pre and MD360 Post. a. USV number in 5 min. A significant increase was found in post-separation USVs on P1, P3, P7 and P8. b. Total duration of USVs. The mice in the MD360 Post group vocalized longer than the mice in the MD360 Pre group on P1, P3 and P7. c. Average USV frequency. A difference in USV frequency between the MD360 Pre and MD360 Post groups was found on P3. *P<0.05, **P<0.01, *** P<0.0001.

Comparison between MD180 Pre and MD180 Post USVs showed significant increases in post-separation USVs in terms of USV number on P1, P3, P7 and P8 (Table 3A); MD180 Post pups called for longer durations than pre-separation USV pups on P3, P7 and P8 (Table 3B); on P14, a decrease in USV frequency was detected in MD180 Post pups (Table 3C) (Fig 5). The comparison between MD360 Pre and MD360 Post suggested that MD360 Post mice called more frequently and for longer durations than MD360 Pre pups on P1, P3 and P7 (Table 4A and 4B); the frequency of USVs in MD360 Post was lower than that in MD360 Pre on P3 (Table 4C) (Fig 6).

**Table 3:** Comparison of USV between MD180 Pre vs. MD180 Post.

**a.USV number**		
Age Group	Results of group comparison	
	*F*_(1,690)_	*P*
P1 MD180 Pre vs. MD180 Post	5.60	0.0182 *
P3 MD180 Pre vs. MD180 Post	13.05	0.0003 ***
P7 MD180 Pre vs. MD180 Post	25.74	<0.0001***
P8 MD180 Pre vs. MD180 Post	14.07	0.0002 ***
P14 MD180 Pre vs. MD180 Post	0.34	0.5604
**b.USV duration**		
Age Group	Results of group comparison	
	*F*_(1,690)_	*P*
P1 MD180 Pre vs. MD180 Post	2.97	0.0853
P3 MD180 Pre vs. MD180 Post	6.53	0.0108*
P7 MD180 Pre vs. MD180 Post	17.29	<0.0001***
P8 MD180 Pre vs. MD180 Post	6.98	0.0084**
P14 MD180 Pre vs. MD180 Post	0.12	0.7276
**c.USV frequency**		
Age Group	Results of group comparison	
	*F*_(1,690)_	*P*
P1 MD180 Pre vs. MD180 Post	0.03	0.8600
P3 MD180 Pre vs. MD180 Post	0.42	0.5156
P7 MD180 Pre vs. MD180 Post	3.27	0.0714
P8 MD180 Pre vs. MD180 Post	0.67	0.4133
P14 MD180 Pre vs. MD180 Post	6.77	0.0096*

*P<0.05

** P<0.01

*** P<0.001

**Table 4:** Comparison of USV between MD360 Pre vs. MD360 Post.

**a. USV number**		
Age Group	Results of group comparison	
	*F*_(1,690)_	*P*
P1 MD360 Pre vs. MD360 Post	9.03	0.0027**
P3 MD360 Pre vs. MD360 Post	84.52	<0.0001***
P7 MD360 Pre vs. MD360 Post	16.63	<0.0001***
P8 MD360 Pre vs. MD360 Post	4.71	0.0304*
P14 MD360 Pre vs. MD360 Post	0.09	0.7657
**b.USV duration**		
Age Group	Results of group comparison	
	*F*_(1,690)_	*P*
P1 MD360 Pre vs. MD360 Post	10.24	0.0014**
P3 MD360 Pre vs. MD360 Post	100.17	<0.0001***
P7 MD360 Pre vs. MD360 Post	12.52	0.0004***
P8 MD360 Pre vs. MD360 Post	1.50	0.2206
P14 MD360 Pre vs. MD360 Post	0.07	0.7934
**c.USV frequency**		
Age Group	Results of group comparison	
	*F*_(1,690)_	*P*
P1 MD360 Pre vs. MD360 Post	0.21	0.6485
P3 MD360 Pre vs. MD360 Post	11.97	0.0006**
P7 MD360 Pre vs. MD360 Post	1.93	0.1658
P8 MD360 Pre vs. MD360 Post	0.03	0.8634
P14 MD360 Pre vs. MD360 Post	1.78	0.1832

*P<0.05

** P<0.01

*** P<0.001

### Sex differences in USV emission

**Fig 7:**
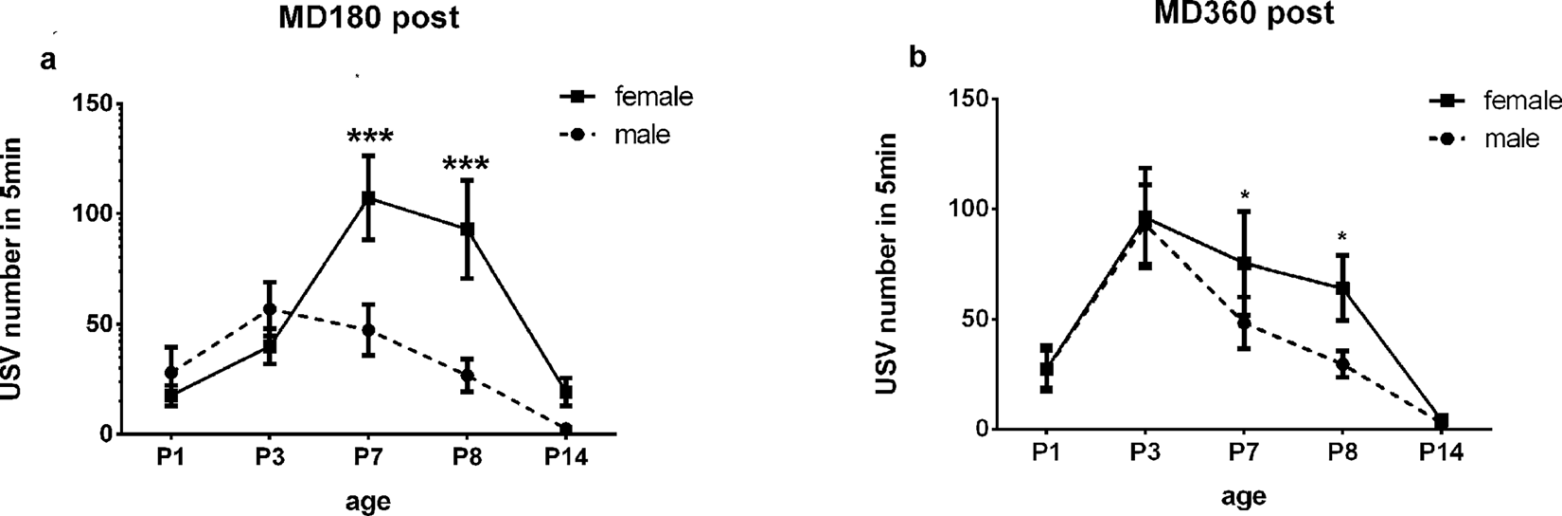
Sex difference in USV number. Female pups called more frequently than male pups on P7 and P8 in the MD180 Post (a) and MD360 Post groups (b). *P<0.05, **P<0.01, *** P<0.0001.

**Fig 8:**
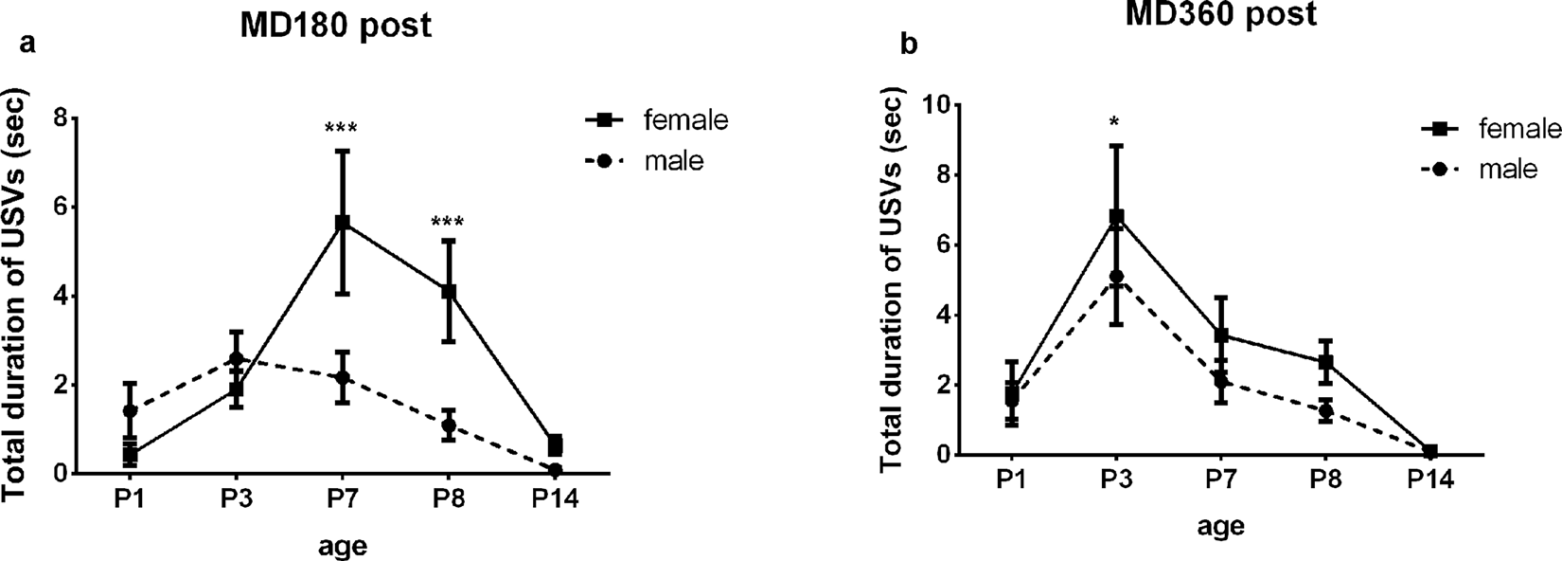
Sex difference in USV duration. Female pups called for longer time than males on P7and P8 in MD180 Post group (a), and on P3 in MD360 group (b). *P<0.05, **P<0.01, *** P<0.0001.

A sex effect was not found in terms of USV number and USV duration for AFR, MD180 Pre and MD360 Pre pups (S2 and S3 Tables). Interestingly, we found significant differences in USV numbers between female and male pups in MD180 Post and a trending difference in MD360 Post (Table 5A), and post hoc testing revealed that females called more frequently than male pups on P7 and P8 in the MD180 Post and MD360 Post groups (Table 6A, Fig 7). USV duration in the MD180 Post and MD360 Post groups was also affected by sex (Table 5B): post hoc testing revealing a similar trend between female and male pups in terms of USV number on P7 and P8 in MD180 Post pups, while a sex difference was found on P3 for MD360 pups (P = 0.0281) (Table 6B, Fig 8). Sex did not influence the USV frequency of pups in all five groups (S4 and S5 Tables).

**Table 5:** Sex differences in USV of MD180 Post and MD360 Post pups.

**a. USV number**		
Group	Male vs. female	
	*F*_(1,690)_	*P*
MD180 Post	11.30	0.0008**
MD360 Post	3.81	0.0512
**b. USV duration**		
Group	Male vs. female	
	*F*_(1,690)_	*P*
MD180 Post	8.08	0.0046**
MD360 Post	4.61	0.0321*

*P<0.05

** P<0.01

**Table 6:** Sex differences in USVofMD180 Post and MD360 Post pups on testing days.

**a. USV number**				
Age	Male vs. female			
	MD180 Post		MD360 Post	
	*F*_(1,690)_	*P*	*F*_(1,690)_	*P*
P1	0.70	0.4022	0.02	0.8946
P3	1.88	0.1713	0.40	0.5257
P7	21.82	<0.0001***	4.19	0.0410*
P8	26.86	<0.0001***	5.90	0.0154*
P14	1.68	0.1953	0.03	0.8630
**b. USV duration**				
Age	Male vs. female			
	MD180 Post MD360 Post			
	*F*_(1,690)_	*P*	*F*_(1,690)_	*P*
P1	1.50	0.2211	0.01	0.9139
P3	0.50	0.4782	5.29	0.0218*
P7	21.03	<0.0001***	1.96	0.1618
P8	16.07	<0.0001***	2.77	0.0967
P14	0.52	0.4730	<0.01	0.9537

*P<0.05

*** P<0.001

## Discussion

### Developmental profile of AFR pup USVs

AFR mice were reared understandard conditions without MD, but there was still isolation stress that can induce USV emission when the AFR pup was individually isolated in the chamber for USV collection. According to previous studies, we can use this 5-min USV detection paradigm to evaluate the USV response to isolation stress. The USV rate of most mouse strains in response to isolation stimuli, such as CS-1, CD-1 and C3H, has been reported to follow a similar ontogenetic profile, with an increase in the first 5–6 days of life, a peak around P6-7, a drop and then near absence at the end of the second postnatal week [36]. However, previous studies of USV development in the C57BL/6 strain have produced mixed results; for instance, Bell et al. reported that in C57BL/6 mice, USV number peaks on P3 [37], whereas Wiaderkiew suggested that the USV rate peaked on P6 and P10, with a decrease on P8 [33]. Our data were consistent with the reported profile of most mouse strains, with a peak at P8. Because USVs are highly strain-specific and sensitive to environmental conditions, the discrepancies in results might be due to differences in the instruments or methods used to detect USVs; for example, in Wiaderkiew’s research, a 7-min detection period was carried out with Avisoft Bioacoustics CM16/CMPA and the Ultrasound Gate recording interface as the USV detector and recording system. In addition, differences between C57BL/6 substrains (e.g., C57BL/6J or C57BL/6N) [38] were not typically considered in prior association studies, but these differences could also contribute to the differences in mixed reports of USV responses to stress. An age effect was also found for the average peak frequency. As shown in Fig 2B, on P7 and P8, a significant increase in average peak frequency was detected. However, we did not detect a significant effect of age on USV duration; although an increase was detected on P8 compared to P1, this increase was different from our expectation and previous reports. According to our results,P3-8 maybe an important period with high isolation-induced USV activity for C57BL/6J mice because all detected peaks and significant differences shown in Table 1 existed during this period. Therefore, P3-8 might be a potential time window for USV analysis for C57BL/6J mice in further studies.

### Effect of MD on USVs

In contrast to our original expectations, MD did not affect pre-separation USVs. Ample evidence has already suggested that USV emissions by infant rodents could be strongly influenced by stressful situations [36,39,40]. For instance, daily social isolation of rat pups for 6 h between P2-7 can result in decreased USV emission at P8 [24]. Similar to the findings of studies conducted with rats, repeated early deprivation could also lead to a decrease in the USV rate of mandarin vole pups [26]. Thus, it is reasonable to expect that the pre-separation USVs of mice might be influenced by repeated MD; however, our data suggested otherwise. Post-separation USVs were found to be robustly affected by MD, as demonstrated by a significant post-MD change in USV number, total duration and average peak frequency. This result demonstrated the interesting finding that the effect of MD on USVs was acute: previous MD experience did not influence the subsequent USV response to isolation stress, indicating the absence of a cumulative influence. To the best of our knowledge, previous studies targeting isolation-induced USVs often used a 5-min isolation test during which the pup was placed in the chamber away from the dam and littermates for USV detection over a period of 5 min, similar to the AFR pups in our study. Considering that USVs may be closely associated with parental absence, thermoregulation, nutritional status and anxiety as previously indicated [26], repeated MD is a significantly different paradigm compared to the 5-min isolation test. Although several studies of rat USVs suggested that repeated MD influences USV emission, we know little about the effects of MD on pup USVs and whether this effect is acute or cumulative in C57BL/6J mice. Our study aims to solve this problem.

However, we cannot exclude the possibility that the pups suffered from a weak physiological condition, such as poor nutrition and lower temperature after MD, although we tried to keep them warm while they were isolated. Alberts JR suggested that rodent pups cannot automatically regulate their body temperature to compensate for their blindness, hairlessness, low subcutaneous fat, and limited motor function [41]; their body temperature rapidly decreases when they are removed from their mother and littermates [32]. A large body of literature has regarded hypothermia as a powerful factor in the regulation of USV emission [42,43]; for example, an increase in USV rate was observed when pups were subjected to isolation combined with a mild cold stimulus [44]. Therefore, the MD-related increase in the number and total duration of post-separation USVs might overlap with the influence of a weak physiological condition. However, it is technically difficult to test this hypothesis because handling and weak physiological status are shared secondary features of MD [28], and we cannot isolate the degree of influence each has on USVs. The interpretation of the results might be further confounded by the fact that maternal behaviors were not well controlled in our study. Given that USVs can be modulated by maternal behaviors [45], it is possible that maternal behavior towards pups after isolation can, to some extent, counteract the effects of MD on USVs; if this is the case, the finding of an acute rather than a cumulative effect of MD seems reasonable. Future work that targets infant-mother interactions and strict physiological control may help elucidate the difference between pre- and post-separation USVs.

Comparison between pre- and post-separation USVs showed significant differences in USV number, duration and frequency between pups in pre and post groups. These results indicated that the effect of MD on USVs of pups after long-term isolation is more robust than the effect before isolation. Past studies suggested that USV is a signal that is sensitive to stress, and some studies even proposed USV as an index of the innate state of rodents [46]. These results added evidence to the reports that USV is a response to stress for infant rodents.

### Decrease in USV frequency after MD

It is important to note that the average peak frequency of USVs in the post-separation group was reduced after MD compared with USVs of AFR mice between P3-P8. Evidence suggests that the prosodic features of USVs, such as frequency and amplitude, can convey information important for emotional development and communication [47]. For example, 22-kHz and 50-kHz USVs have been shown to be closely related to positive and negative states in rats [48,49]. However, the relationship between USV frequency and emotional state in mice is not yet well understood and is actively being explored. To the best of our knowledge, past studies of USV frequency have largely, if not exclusively, used knockout pups or adult mice to investigate the role of USVs or related genes in social communication [50–52]; whether neonatal stress can affect USV frequency in mouse pups has rarely been considered. Our data suggesting a decrease in USV frequency after MD provides a rudimentary understanding of the possible relationship between frequency and stress and indicates that the frequency of USVs emitted by mouse pups might be a novel index that can be affected by stressful events in early life.

### Sex differences in USV emission

Although sex differences in auditory communication have been suggested to be common in animals [53], particularly in the context of attracting mating patterns [30,40], differences in USV emission under stress between males and females have been less frequently investigated in mouse pups. In our study, USV emission was sexually dimorphic when the age effect was taken into account in MD180 Post and MD360 Post pups; we found that the female pups called more frequently and for longer durations than the males on P7 and P8 in the MD180 Post group, and the females vocalized for a longer period of time on P3 than the males among MD360 Post pups; no obvious differences were found on the other test days and in terms of USV frequency. Our data indicated that female pups were more susceptible than males to the effect of MD on post-separation USV number and duration.

Although the mechanism underlying the sex effect and the significant age X sex interaction was not the focus of the present study, we hypothesize that maternal factors and sample size may have contributed to these results [54]. It was suggested that a sex-specific bias in maternal care is evident in rats; for example, dams prefer to groom male pups at a higher rate than females. Thus, there is a possibility that this sex bias also exists in mouse pups, which leads to sex differences in the USV response to MD. USVs are a highly variable parameter [55], and it is possible that we only found a sex difference during the period with high USV activity due to an insufficient sample size. Therefore, it will be intriguing to further investigate the effect of sex on USV development with a larger sample size. If USV is a predictor of affective state as proposed [20], this result might provide valuable information about sex differences in emotional development under conditions of acute stress in C57BL/6J mice [54].

### The methodological limitations of USV detection

Although our study produced information about USVs, these results may be influenced by the resolution of the USV detector used in our study. The Med ANL-937-1 detector, which has a temporal resolution of 30ms, is not an ideal USV detector for the analysis of pup USVs, which are very short in duration; for example, Branchi et al. reported that isolation-induced USVs have a duration of 10-200ms [36]. Evidence has shown that the USV duration of rodents can be increased by stressful conditions [26], which makes it more likely that USVs emitted under stressful conditions may be more easily detected than USVs emitted under normal conditions when a temporal resolution of 30ms is used. However, we cannot rule out this possibility using our USV detector. For the same reason, we could not thoroughly analyze the detailed acoustic parameters of the USVs, such as the number of USV bouts [33] and syllable types, which have the characteristics of songs and convey important information for communication [47]. Further studies using a high-resolution USV detector will help us more fully understand and appreciate the USV changes that occur in a stressful environment.

## Conclusion

The present study provides a deeper description of the developmental profile of USVs by exploring how infant C57BL/6J mice respond to early life stress based on measurements of USV emissions. Our data extends previous observations because we investigated sex differences and whether the effect of MD was acute or cumulative. Prior studies have suggested that the C57BL/6 strain is “stress resistant” [28]. Based on our USV results, it is not clear whether the absence of cumulative influence could be partly due to this resistance; thus, further evidence is needed to test this hypothesis and to explore the mechanism underlying sex differences. In particular, such explorations should be combined with investigations of the stress-related neurological changes that occur in the periaqueductal gray, which plays an important role in USV emission [56]. Although the results of our study are preliminary due to methodological limitations, they still provide information to aid in the interpretation of the USV response to life stress and underscore the necessity of considering sex differences in the USV emissions of very young C57BL/6J mice.

## Supporting Information

S1 Table The effect of MD on pre-separation USVs. No significant differences were found between the USVs of AFR, MD180 Pre and MD360 Pre mice on USV number, duration and frequency.
(DOCX)

S2 Table Sex difference on USV in AFR, MD180 Pre and MD360 Pre pups. The effect of sex was not significant on USV number and duration between these groups.
(DOCX)

S3 Table Sex difference on USV in AFR, MD180 Pre and MD360 Pre pups on testing days. No sexual effect was found on USV number and duration between these groups on all testing days.
(DOCX)

S4 Table Sex difference on USV frequency in five groups. Sex effect was not significant on USV frequency for all the groups in the study.
(DOCX)

S5 Table Sex difference on USV frequency in five groups on testing days. No sexual effect was found on USV frequency between the five groups on all testing days.
(DOCX)
