# Supplementary material for: Maternal Deprivation Influences Pup Ultrasonic Vocalizations of C57BL/6J Mice
Source: PLoS One. 2016 Aug 23;11(8):e0160409. doi: 10.1371/journal.pone.0160409 (PMC4994965; doi:10.1371/journal.pone.0160409)
Supplement: S1 Table — No significant differences were found between the USVs of AFR, MD180 Pre and MD360 Pre mice on USV number, duration and frequency. (DOCX) [file pone.0160409.s001.docx]

**S1 Table** MD effect on USV of AFR, MD180Pre and MD360Pre pups

| **a. USV number** | | | | |
| --- | --- | --- | --- | --- |
| Age Group | | Results of Group comparison | | |
|  |  | *F* | | *P* |
| P1 AFR vs MD180Pre | | 0.00 | | 0.9951 |
| AFR vs MD360re | | 0.01 | | 0.9388 |
| MD180Pre vs MD360Pre | | 0.00 | | 0.9439 |
| P3 AFR vs MD180Pre | | 0.26 | | 0.6134 |
| AFR vs MD360Pre | | 0.39 | | 0.5303 |
| MD180Pre vs MD360Pre | | 1.33 | | 0.2494 |
| P7 AFR vs MD180Pre | | 1.43 | | 0.2321 |
| AFR vs MD360Pre | | 0.19 | | 0.6660 |
| MD180Pre vs MD360Pre | | 0.62 | | 0.4303 |
| P8 AFR vs MD180pre | | 0.03 | | 0.8672 |
| AFR vs MD360Pre | | 0.00 | | 0.9615 |
| MD180Pre vs MD360Pre | | 0.05 | | 0.8231 |
| P14 AFR vs MD180Pre | | 0.00 | | 0.9779 |
| AFR vs MD360Pre | | 0.01 | | 0.9336 |
| MD180Pre vs MD360Pre | | 0.00 | | 0.9565 |
| **b. USV duration** | | | | |
| Age Group | | Results of Group comparison | | |
|  |  | *F* | *P* | |
| P1 AFR vs MD180Pre | | 0.00 | 0.9987 | |
| AFR vs MD360re | | 0.00 | 0.9514 | |
| MD180Pre vs MD360Pre | | 0.00 | 0.9501 | |
| P3 AFR vs MD180Pre | | 0.35 | 0.5570 | |
| AFR vs MD360Pre | | 0.17 | 0.6829 | |
| MD180Pre vs MD360Pre | | 1.04 | 0.3086 | |
| P7 AFR vs MD180Pre | | 2.03 | 0.1545 | |
| AFR vs MD360Pre | | 0.01 | 0.9355 | |
| MD180Pre vs MD360Pre | | 1.89 | 0.1698 | |
| P8 AFR vs MD180pre | | 0.01 | 0.9347 | |
| AFR vs MD360Pre | | 0.06 | 0.8003 | |
| MD180Pre vs MD360Pre | | 0.03 | 0.8656 | |
| P14 AFR vs MD180Pre | | 0.00 | 0.9840 | |
| AFR vs MD360Pre | | 0.00 | 0.9853 | |
| MD180Pre vs MD360Pre | | 0.00 | 0.9687 | |
| **c. USV frequency** |  | | | |
| Age Group | Results of Group comparison | | | |
|  | *F* | | *P* | |
| P1 AFR vs MD180Pre | | 0.00 | 0.9919 | |
| AFR vs MD360re | | 0.79 | 0.3751 | |
| MD180Pre vs MD360Pre | | 0.55 | 0.4584 | |
| P3 AFR vs MD180Pre | | 0.05 | 0.8257 | |
| AFR vs MD360Pre | | 1.66 | 0.1988 | |
| MD180Pre vs MD360Pre | | 2.35 | 0.1257 | |
| P7 AFR vs MD180Pre | | 3.91 | 0.0486 | |
| AFR vs MD360Pre | | 5.39 | 0.0207 | |
| MD180Pre vs MD360Pre | | 0.09 | 0.7609 | |
| P8 AFR vs MD180pre | | 2.09 | 0.1488 | |
| AFR vs MD360Pre | | 2.82 | 0.0937 | |
| MD180Pre vs MD360Pre | | 0.05 | 0.8180 | |
| P14 AFR vs MD180Pre | | 0.05 | 0.8257 | |
| AFR vs MD360Pre | | 0.11 | 0.7414 | |
| MD180Pre vs MD360Pre | | 0.25 | 0.6139 | |
