# Supplementary material for: Maternal Deprivation Influences Pup Ultrasonic Vocalizations of C57BL/6J Mice
Source: PLoS One. 2016 Aug 23;11(8):e0160409. doi: 10.1371/journal.pone.0160409 (PMC4994965; doi:10.1371/journal.pone.0160409)
Supplement: S2 Table — The effect of sex was not significant on USV number and duration between these groups. (DOCX) [file pone.0160409.s002.docx]

**S2 Table** Sex difference on USV in AFR, MD180Pre and MD360Pre pups

| **a. USV number** | | |
| --- | --- | --- |
| Groups | Male *vs* Female | |
|  | *F* | *P* |
| AFR | 0.48 | 0.4866 |
| MD180Pre | 0.03 | 0.8693 |
| MD360Pre | 0.13 | 0.7148 |
| **b. USV duration** | | |
| Groups | Male *vs* Female | |
|  | *F* | *P* |
| AFR | 0.35 | 0.5531 |
| MD180Pre | <0.01 | 0.9766 |
| MD360Pre | 0.08 | 0.7552 |
