# Supplementary material for: Maternal Deprivation Influences Pup Ultrasonic Vocalizations of C57BL/6J Mice
Source: PLoS One. 2016 Aug 23;11(8):e0160409. doi: 10.1371/journal.pone.0160409 (PMC4994965; doi:10.1371/journal.pone.0160409)
Supplement: S3 Table — No sexual effect was found on USV number and duration between these groups on all testing days. (DOCX) [file pone.0160409.s003.docx]

**S3 Table** Sex difference on USV in AFR, MDPre and MD360Pre pups on testing days

| **a. USV number** | | | | | | |
| --- | --- | --- | --- | --- | --- | --- |
| Age | Male *vs* Female | | | | | |
|  | AFR | | MD180Pre | | MD360Pre | |
|  | *F* | *P* | *F* | *P* | *F* | *P* |
| P1 | <0.01 | 0.9659 | <0.01 | 0.9978 | <0.01 | 0.9531 |
| P3 | 0.37 | 0.5431 | 0.44 | 0.5081 | 0.13 | 0.7193 |
| P7 | 0.86 | 0.3541 | 0.01 | 0.9410 | 0.03 | 0.8662 |
| P8 | 0.25 | 0.6163 | 2.51 | 0.1137 | 0.62 | 0.4323 |
| P14 | 0.05 | 0.8270 | 0.31 | 0.5751 | 0.02 | 0.8990 |
| **b. USV duration** | | | | | | |
| Age | Male *vs* Female | | | | | |
|  | AFR MD180Pre MD360Pre | | | | | |
|  | *F* | *P* | *F* | *P* | *F* | *P* |
| P1 | <0.01 | 0.9759 | <0.01 | 0.9969 | <0.01 | 0.9633 |
| P3 | 0.35 | 0.5518 | 0.40 | 0.5259 | 0.10 | 0.7533 |
| P7 | 0.49 | 0.4824 | 0.18 | 0.6702 | 0.10 | 0.7524 |
| P8 | 0.13 | 0.7185 | 1.67 | 0.1965 | 0.45 | 0.5027 |
| P14 | 0.02 | 0.8926 | 0.10 | 0.7550 | <0.01 | 0.9510 |
