# Supplementary material for: Maternal Deprivation Influences Pup Ultrasonic Vocalizations of C57BL/6J Mice
Source: PLoS One. 2016 Aug 23;11(8):e0160409. doi: 10.1371/journal.pone.0160409 (PMC4994965; doi:10.1371/journal.pone.0160409)
Supplement: S4 Table — Sex effect was not significant on USV frequency for all the groups in the study. (DOCX) [file pone.0160409.s004.docx]

**S4 Table** Sex difference on USV frequency in five groups

| **Group** | **Male *vs* Female** | |
| --- | --- | --- |
|  | ***F*** | ***P*** |
| AFR | 0.23 | 0.6353 |
| MD180Pre | 0.19 | 0.6601 |
| MD180Post | 0.50 | 0.4794 |
| MD360Pre | 0.03 | 0.8666 |
| MD360Post | 0.22 | 0.6411 |
