# Supplementary material for: Maternal Deprivation Influences Pup Ultrasonic Vocalizations of C57BL/6J Mice
Source: PLoS One. 2016 Aug 23;11(8):e0160409. doi: 10.1371/journal.pone.0160409 (PMC4994965; doi:10.1371/journal.pone.0160409)
Supplement: S5 Table — No sexual effect was found on USV frequency between the five groups on all testing days. (DOCX) [file pone.0160409.s005.docx]

**S5 Table** Sex difference on USV frequency in five groups on testing days

| **Age** | **Male *vs* Female** | | | | | | | | | |
| --- | --- | --- | --- | --- | --- | --- | --- | --- | --- | --- |
|  | **AFR** | | **MD180Pre** | | **MD180Post** | | **MD360Pre** | | **MD360Post** | |
|  | ***F*** | ***P*** | ***F*** | ***P*** | ***F*** | ***P*** | ***F*** | ***P*** | ***F*** | ***P*** |
| P1 | 0.33 | 0.5683 | 0.02 | 0.8927 | 2.50 | 0.1149 | 0.24 | 0.6226 | 0.01 | 0.9031 |
| P3 | 0.04 | 0.8513 | 0.01 | 0.9206 | 0.25 | 0.6205 | 1.02 | 0.3130 | 0.57 | 0.4507 |
| P7 | 0.99 | 0.3194 | 0.19 | 0.6668 | 2.11 | 0.1466 | 0.05 | 0.8303 | 0.56 | 0.4542 |
| P8 | 0.12 | 0.7304 | 0.38 | 0.5374 | 1.34 | 0.2470 | 2.85 | 0.0921 | 0.90 | 0.3425 |
| P14 | 0.91 | 0.3401 | 2.77 | 0.0971 | 1.13 | 0.2894 | 1.64 | 0.2009 | 0.11 | 0.7366 |
